# Ferroelectric memory based on nanostructures

**DOI:** 10.1186/1556-276X-7-285

**Published:** 2012-06-01

**Authors:** Xingqiang Liu, Yueli Liu, Wen Chen, Jinchai Li, Lei Liao

**Affiliations:** 1 Key Laboratory of Artificial Micro- and Nano-structures of Ministry of Education, and School of Physics and Technology, Wuhan University, Wuhan, 430072, People's Republic of China; 2 State Key Laboratory of Advanced Technology for Materials Synthesis and Processing, and School of Materials Science and Engineering, Wuhan University of Technology, Wuhan, 430070, People's Republic of China

## Abstract

In the past decades, ferroelectric materials have attracted wide attention due to their applications in nonvolatile memory devices (NVMDs) rendered by the electrically switchable spontaneous polarizations. Furthermore, the combination of ferroelectric and nanomaterials opens a new route to fabricating a nanoscale memory device with ultrahigh memory integration, which greatly eases the ever increasing scaling and economic challenges encountered in the traditional semiconductor industry. In this review, we summarize the recent development of the nonvolatile ferroelectric field effect transistor (FeFET) memory devices based on nanostructures. The operating principles of FeFET are introduced first, followed by the discussion of the real FeFET memory nanodevices based on oxide nanowires, nanoparticles, semiconductor nanotetrapods, carbon nanotubes, and graphene. Finally, we present the opportunities and challenges in nanomemory devices and our views on the future prospects of NVMDs.

## Introduction

According to Moore's law, the number of transistors accommodated on the integrated circuits doubles roughly every 18 months and so does the performance [1]. As the essential part of the integrated circuits, nonvolatile memory devices (NVMDs) have been heavily deployed in portable electronic devices to realize secure and fast data transfer, such as the ID cards, MP3 player, and so on. The versatile NVMDs should be reprogrammable and require a mechanism of repeatable switching between different binary states [2-4]. The ferroelectric field effect transistor (FeFET) is one of such promising NVMDs with the lowest power consumption [5] and high speed bearing comparable to that of dynamic random access memory [6]. Other memory mechanisms including polarization induced by the polar molecule (such as H_2_O) adsorption/desorption and by the defect-related charge-trapping layer have also been studied [7-9]. However, both the adsorption/desorption and the defect-related charge-trapping mechanisms suffer from reproducibility problems caused by the nature that neither the adsorption/desorption of polar molecules nor the amount or distribution of the defects can be exactly controlled, which creates a great challenge for reproduction. This review therefore gives an overview of the advances of FeFET for NVMDs in the current state and the future.

The simple architectural structures and mature fabrication technologies of the traditional thin-film transistor have sparked a surge of interest in the thin-film FeFET for NVMDs. However, theoretical calculation has shown that the planar corrugations effectively worsen the distribution of polarization bound charges [10], due to smearing of the phase transition. It's well recognized that the physical properties of ferroelectric thin film are significantly limited by a critical size [11-13]. Furthermore, with the decrease in the thickness of the ferroelectric thin film, the remnant polarization (*P*_r_) decreases and the coercive field (*E*_c_) turns up increasingly due to the collapsed dielectric response [14-18]. This imposes a serious limitation on the desired integrated density and leads to poor performance in the thin-film transistor-based NVMDs [19]. In order to fulfill the particularly required performance such as retention time, endurance, response time, and/or power consumption, plenty of nanomaterials and alternative technologies have been utilized to enhance the integrated density and performance, which open a route to overcome the scaling limitations and economic challenges encountered in the current silicon industry [20-24]. In this survey, we summarize the current researches on fabricating a promising nano-FeFET. This paper is organized as follows: the ‘Ferroelectric and the operating principle of FeFET’ section summarizes the structure and characters of ferroelectric and the operating principle of FeFET. The ‘Current researches’ section reviews the current state of nano-FeFET devices, including the combinations of ferroelectrics with nanowires (NWs) [17,25-28], nanoparticles (NPs) [29-32], three-dimensional (3D) nanostructures [33-35], carbon nanotubes (CNTs) [36-45], and graphene [43,46-49]. The ‘Challenges and improvements’ section explains the fatigue mechanism and provides an overview of the efforts that have been taken to improve the fatigue resistance. The ‘Conclusions’ section gives an outlook and conclusion for the practical applications of FeFET.

### Ferroelectric and the operating principle of FeFET

The uniform characters of ferroelectrics offer opportunities for fabricating NVMDs. Devices based on one-dimensional (1D) [17,25,37-39] or two-dimensional (2D) [43,46] nanostructures have been realized with excellent performance [50,51]. This section is divided into two parts: Ferroelectric is introduced first followed by the descriptions of the structure and principle of polarization. The operating and programming principles of FeFET for memory are then presented.

#### *Ferroelectric*

**Figure 1:**
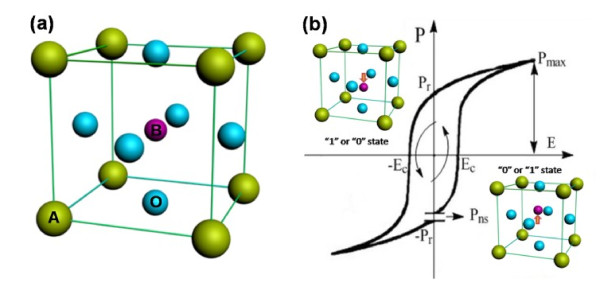
**Schematic structure (a) and hysteresis loop curve of ferroelectric (b).** In **(a)**, the green balls represent the A^2+^, blue balls are O^2−^, and the purple one is B^4+^; they are located at the vertex angle, centroid, and body center, respectively. In **(b)**, the inserts are the corresponding location of B^4+^ in the lattice cell, with the bistability state obviously different from that of the dynamic random access memory which needs power to maintain its state.

In general, ferroelectrics are dielectric crystals with the perovskite structure, [22] whose formula is ABO_3_ with the schematic structure shown in Figure 1a. The spontaneous polarization arises as the temperature sweeps due to a lattice distortion which involves the relative displacements of B^4+^ in each cell. These ferroelectric behaviors appear only under an inherent temperature (Curie temperature *T*_C_). As shown in Figure 1b, the similar polarization phenomenon can also be exhibited from the ferroelectric under the condition of an external electric field (*E*), in which the intensity of polarization (*P*) does not exhibit a linear response to *E* but instead shows a closed hysteretic loop. When *E* strides a particular value, the polarization is reversed, and we call this value coercive field *E*_c_ (see Figure 1b). In other words, *P* can be switched by modulating *E*, and the remnant polarization + *P*_r_ and − *P*_r_ states are stored in ferroelectric. The bistable state of ferroelectric can be programmed as binary information ‘1’ and ‘0’ for NVMDs. Considerable efforts therefore have been taken to exploit available NVMD devices based on this.

#### *FeFET*

**Figure 2:**
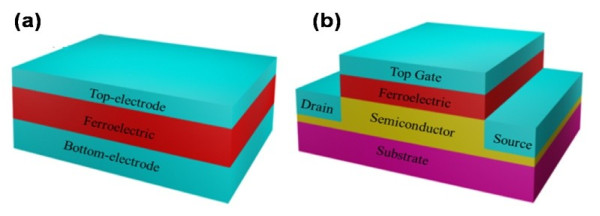
**The current models of NVMDs.** (**a**) The capacitor model, consisting of a thin ferroelectric film between two conductive electrodes. (**b**) The FeFET model, which replaces the dielectric of MOSFET with ferroelectric.

A reasonable model is critical in order to take the advantages of ferroelectric for fabricating the NVMDs [52]. The typical memory devices are built on the base of the capacitor [5,53,54] or FeFET [55,56]. The former model consists of a thin ferroelectric film between two conductive electrodes, and the latter one is similar to a metal-oxide-semiconductor field-effect transistor (MOSFET). Figure 2 shows the schematic diagrams of both. Shiga et al*.* have demonstrated that the signal significantly deteriorates as the capacitor size scales down, which limits the memory capacity to 128 Mb. [57]. The leakage current is another key challenge for the development of capacitor-based NVMDs [58]. On the other hand, FeFET has a well-defined memory switch behavior with simple nondestructive readout (NDRO) process carried out by detecting *I*_DS_ or the resistance of the active channel. Due to the excellent performance obtained, the integration of ferroelectrics with nanomaterials has been extensively explored in previous works [2,43,59-61], where ultimate scalability has been reported as well. Thus, in this paper, we give the special attention to FeFET-based memory devices with nanostructures.

**Figure 3:**
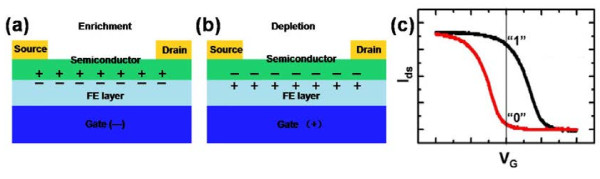
**Schematic views of p-type FeFET and corresponding hysteretic loop of polarization.** (**a**,**b**) Schematic views of p-type FeFET for a simplified field-effect transistor model. (**c**) The corresponding hysteretic loop of polarization varies with the external electric field.

Unlike MOSFET, the oxide-gate dielectric is replaced by ferroelectric in FeFET. By modulating the gate bias, the carriers accumulate or deplete at the ferroelectric (FE)-semiconductor interface, leading the FeFET on or off, as shown in Figure 3a,b (a p-channel FeFET) [62]. The corresponding transfer curve of *I*_DS_ versus *V*_G_ has been depicted in Figure 3c. With the polarization of the FE layer, the curve of *I*_DS_ varies with *V*_G_ as a hysteretic loop when *V*_G_ sweeps upward (from negative to positive) and then downward (from positive to negative) continuously. Moreover, even if *V*_G_ is released, the charges remain; thus, the device retains its state. Therefore, as *V*_G_ = 0 V, the device still exhibits an on or off state, which can be defined as 1 and 0. In other words, the information in FeFET is not lost even when encountering a power outage. The information can be read out by detecting the *I*_DS_ or the resistance of the active channel. Figure 3c is the corresponding closed hysteretic loop of *I*_DS_ versus *V*_G_, which shows the track of switching between the 0 and 1 state. It's evident that the appropriate large value of *P*_r_ and low *E*_C_ are important for the performance of FeFET [59,63]. A too low *P*_r_ may not be able to induce enough accumulated carriers at the FE-semiconductor interface to give rise to an evident change of conductance of the channel. On the other hand, although a low *E*_C_ can realize a low operating bias, it also raises a serious accompanying instability problem since a low voltage is not enough to switch the states of the device, which can be solved by the concessive method using a thick FE layer. In general, the properties of ferroelectric are essential for the fabrication of FeFET.

### Current researches

With the development of the fundamental material science, tremendous progress has been made to fabricate FeFET for NVMDs based on coetaneous advanced materials. In this section, we discuss the current research on FeFET for NVMDs.

#### *Oxide NW-based FeFET*

**Figure 4:**
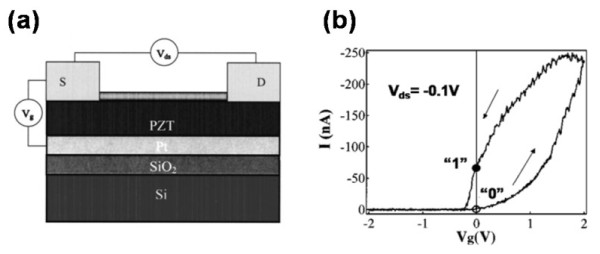
**Schematic circuit diagram of In**_**2**_**O**_**3**_**nanowire FeFET (a) and characteristics of PZT-gated In**_**2**_**O**_**3**_**NW transistor (b).** The PZT-gated In_2_O_3_ NW transistor with *V*_DS_ = −0.1 V shows pronounced hysteresis. ‘1’ and ‘0’ denote two states at *V*_G_ = 0 V for the memory operation.

Based on the traditional thin-film FET, it is easy to fabricate a thin-film FeFET. Although its simple structure can supply easy fabrication [64], it also suffers from several problems which need to be solved [65,66]. For example, the *E*_C_ of the film is typically several kilovolts per centimeter, which requires a high operating voltage to reverse the polarization. Moreover, the poor polarization value can hardly effect an evident conductance change [67]. On top of these, the low field-effect mobility, low on/off ratio, low subthreshold slope, and the poor switch speed are limitations impacting the applications of the thin-film FeFET [68-70]. In recent years, oxide NWs have emerged as promising building blocks in various technological domains including fundamental researches and nanodevice applications due to their unique structures and stable properties [71,72]. Tremendous efforts have been made to fabricate NW FeFETs, which use the NWs as the active channel. In the early days, In_2_O_3_ NWs have been integrated with lead zirconate titanate (PZT) to fabricate FeFET. Due to the high dielectric constant and the switchable spontaneous polarization of PZT, the fabricated device received an enhanced performance and memory effect [25], when compared with the traditional SiO_2_-gate FET. The schematic diagram is shown in Figure 4a, which reveals that the back-gate FeFET structure has been used in this research. The In_2_O_3_ NWs with a diameter of 10 nm were first ultrasonicated in isopropanol and then deposited onto the PZT/Pt/SiO_2_/Si substrate by spin-coating technique. Photolithography, Ti/Au deposition, and lift off were carried out subsequently to pattern the source and drain electrodes, which were in contact with an individual NW. The fabricated FeFET operated on an accumulation/depletion mode with the conduction of the active NW channel modulated by the gate potential. Figure 4b shows the transfer curves of the memory device, exhibiting a closed counterclockwise loop, when *V*_G_ was sweeping upward and then downward. As *V*_G_ = 0 V, there were two pronouncedly different values of *I*_DS_, which were caused by the switchable *P*_r_ of the PZT layer. Hence, we could define the larger one as a binary 1 and the smaller one as 0 to realize the basic program function. Moreover, the methods used in this experiment could be generalized and applied to other NW systems to obtain nanoscale memory devices.

**Figure 5:**
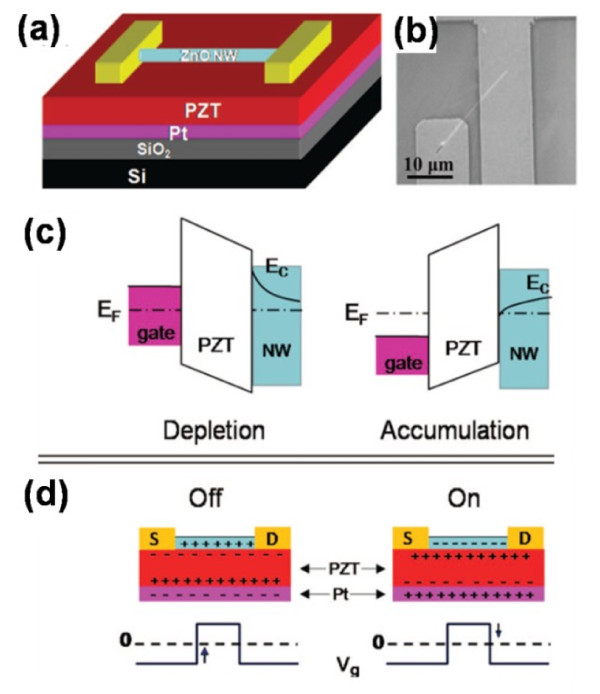
**ZnO NW FeFET device.** (**a**) Schematics of the device configuration. (**b**) SEM image of a single device. (**c**) Corresponding band diagram showing the gate effect. (**d**) Idealized field-effect model of the ZnO NW FeFET device at off and on states, without considering surface and interface trap charges.

**Figure 6:**
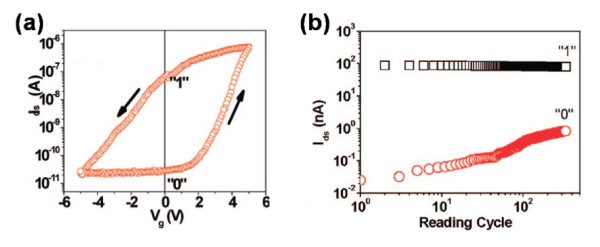
**Memory characteristics ZnO NW FeFET device.** (**a**) *I*_DS_*-V*_G_ transfer characteristics at *V*_DS_ = 2 V of a FeFET device based on a ferroelectric PZT gate oxide. (**b**) Endurance tests by measuring the off- and on-state drain current at a fixed *V*_DS_ = 2 V as a function of programming cycles of the ZnO nanowire-based FET devices.

Having inherent defects in ZnO NWs, such as oxygen vacancies and Zn interstitials, ZnO NWs present the character of the natural n-type semiconductor [73]. In Liao's recent work [2], ZnO NW was combined with PZT thin film to realize the memory function successfully. The schematic diagram and scanning electron microscopy (SEM) image were shown in Figure 5a,b, respectively. As ZnO NW is an n-type semiconductor, with the polarization of PZT, a positive pulse gate voltage would raise up the band of the channel and then deplete the electrons in the NW, as shown in Figure 5c. The device presented an effectively ‘off’ state, which could be defined as a binary 0. The binary 1 representing the opposite state could be defined as well in Figure 5d. Based on this principle, the state of the device could be switched by a timely pulse; in other words, the programming process was realized. The transfer character also demonstrated the switching mechanism with two pronounced states at *V*_G_ = 0 V, which is shown in Figure 6a. Reading cycles of 10^3^ of both 1 and 0 states were carried out in Figure 6b. As we can see, the two states were still well distinguishable, showing a sustaining memory performance.

**Figure 7:**
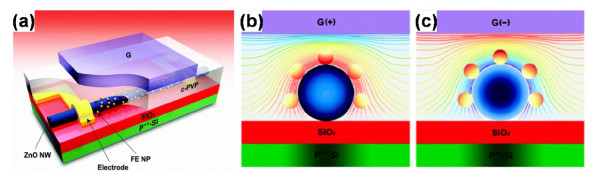
**Schematic views of ZnO NW FET and polarization model for FE NPs surrounding a NW.** (**a**) A schematic view of a top-gate FET-based nonvolatile memory device. For a top-gate ZnO NW FET where a ZnO NW is incorporated with FE NPs, cross-linked poly (4-vinylphenol) (c-PVP) was used as a gate dielectric. (**b**,**c**) The schematic views of a simplified polarization model for FE NPs surrounding a NW. The lines show the electric field distribution between a ZnO NW and a gate electrode. Electric dipole moments are reoriented along electric field lines, resulting in different polarization states according to the gate electric field strength. Furthermore, affected by more reoriented electric dipole moments, the denser the equipotential lines around the upper part of a NW are, the easier it is to induce more polarized charges at the FE-NW interface. Thus, different conductance states result from different net amounts of reoriented electric dipole moments dependent on the applied gate electric field strength.

**Figure 8:**
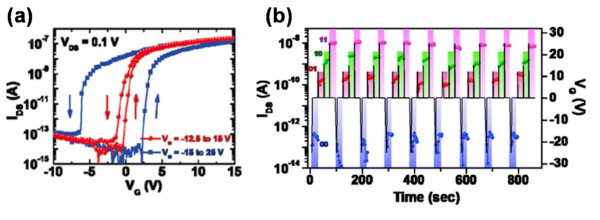
**Hysteresis behaviors of top-gate NW FET and the switching characteristics.** (**a**) Hysteresis behaviors of a top-gate NW FET as a function of the sweep range of gate voltages. Compared with the hysteretic behavior of a back-gate FET with a clockwise hysteresis loop, devices with the top-gate structure show a counterclockwise hysteresis loop, confirming that the origin of such hysteretic behaviors is due to the polarization of FEs. Arrows indicate gate voltage sweep directions. (**b**) Switching characteristics of a device with a top gate structure measured with *V*_DS_ =0.1 V and *V*_G_ = 0 V, clearly showing that a FeFET functions as a two-bit memory with four different conductance states defined as 00, 01, 10, and 11 after the application of gate voltage pulses of −25, +12, +15, and +25 V, respectively.

Despite the achievements made with the NWs, which yield many attractive features and desirable capability for potential applications, there are still many more new approaches coming up to further improve the integrated density. The multi-bit FeFET has been considered to supply higher density for storage, which could overcome the scaling limitations and economic challenges in the current silicon industry. The ZnO NW FET (Figure 7a) with coated ferroelectric BaTiO_3_ (BTO) NPs has realized the function for a two-bit memory [17]. Figure 7b,c showed the schematic view of the differing degrees of reoriented electric dipole moments when a positive and negative bias was applied, respectively. The polarization of the NPs gave rise to a higher positive gate bias and then induced more polarized NPs. The more polarized charges were accumulated at the NP-NW interface, the larger the conductance of the active channel was. The surface engineering further caused a positive shift of the threshold voltage (*V*_th_). In Figure 8a, it's obvious to find that *V*_th_ was dependent on the sweep range of the gate voltage as well as the sweep direction. A more negative initial sweep voltage caused a more positive shift of *V*_th_. Similarly, a more negative threshold voltage shift was related to a more positive initial sweep voltage. In addition, as mentioned previously, the information could be read out by detecting the current or the active channel resistance, so an individual device used herein could store multi-bit information with different gate voltage pulses. The different states of the *I*_DS_ were corresponding to different binary information, which could be modulated by varying gate voltage pulses. Figure 8b showed the detailed practical process of programming ‘00’, ‘01’, ‘10’, and ‘11’ states. With the ability of storing multi-bit information in an individual memory device, the device reported herein provided a new way to enhance the integrated density.

Furthermore, the synthesis methods applied here demonstrated a simple room-temperature process for integrating the FE NPs with ZnO NW to fabricate the multi-bit memory device. The device fabricated in this way had a remarkably high on/off ratio of 10^4^ and a long retention time over 4 × 10^4^ s, which made it easy to recognize the two binary states. This work thus provided a viable route to fabricate high density NVMDs to overcome the existing physical and technological limitations.

#### *Nanotetrapod-based FeFET*

In order to exploit the bottom-up technology, extensive studies on 3D structure-based devices have flourished, inspired by the peculiar prosperity of the architectures. Depending on the kinetics of the growth process, two crystal structures of one same compound can exist stably. Despite the changes in size, the additional structure provides more electronic states and characters. These special features provide the precious opportunity for making efficient nanodevices. CdS nanotetrapods provide a typical example in which each individual nanotetrapod is combined with the pyramidal-shaped zincblende structure core and wurtzite arms, with the electrons and holes located in each other, respectively. Moreover, the bandgap of the arms is larger than the one of the core. With the type II band alignment, a peculiar electron transport is observed.

**Figure 9:**
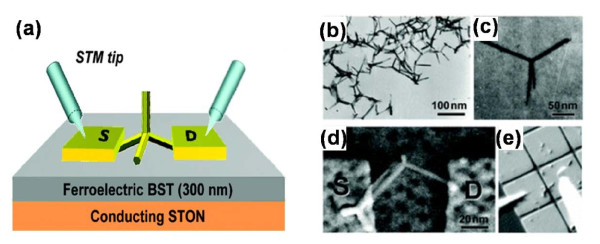
**Schematic illustration of nanotetrapod transistor and images of CdS nanotetrapod and the fabricated device.** (**a**) Schematic illustration of a nanotetrapod transistor with a 300-nm-thick ferroelectric dielectric under testing with scanning tunneling microscope (STM) tips. The source (S) and drain (D) electrodes are the patterned Pt layer. (**b**) Typical TEM image of the multiarmed CdS nanotetrapod used in this study. (**c**) The enlarged micrograph of a single CdS nanotetrapod. (**d**) SEM image of a single CdS nanotetrapod device. (**e**) *In situ* SEM image of two STM tips (shown in white) probing on a testing device.

**Figure 10:**
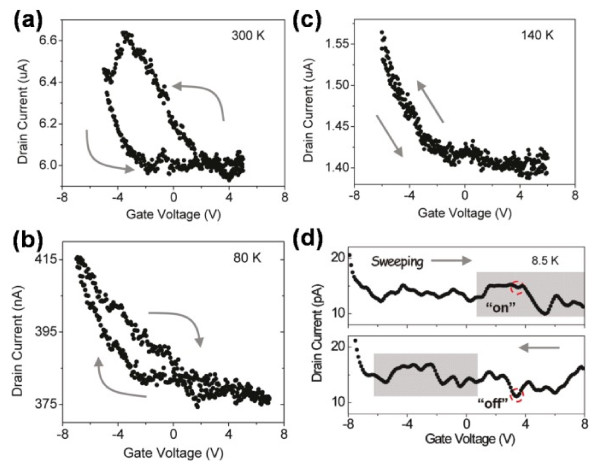
***I*-*V***_**G**_**transfer characteristics.** Typical *I*-*V*_G_ transfer characteristic was measured at (**a**) 300, (**b**) 140, (**c**) 80, and (**d**) 8.5 K (with *V*_DS_ = 2 V for a to c; *V*_DS_ = 50 mV for d). In (**a**), a counterclockwise hysteresis loop occurs at room temperature due to a charge-store effect. In (**b**), at 80 K, a clockwise hysteresis loop is opened, indicating a nonvolatile memory operation. In (**c**), a competition between the ferroelectric effect and the charge-storage effect essentially closes the memory window at 140 K. In (**d**), at 8.5 K, a ferroelectric-modulated SET behavior is observed. The two red circles represent a bistable state. The sharp increase at a gate voltage of −6 V is due to the leakage current.

Due to the unique and also discommodious 3D structure, CdS nanotetrapods were impossible to lie flat on the gate, resulting in poor capacitance coupling, whereas the ferroelectric with high dielectric constant can make up it; therefore, the memory effect was also observed [35]. Figure 9a shows the schematic diagram, Figure 9b,c shows the transmission electron microscopy (TEM) images of CdS nanotetrapods, and Figure 9d,e shows the SEM images of the device. To investigate the performance of the prepared device, the transfer character of the device was measured with the gate voltage swept upward and then downward continually under various temperatures (see Table 1). It is obvious that a counterclockwise hysteretic loop is presented at 300 K, whereas a clockwise hysteretic loop is presented at 80 K. The phenomenon in Figure 1a could be attributed to the ‘charge-store’ memory effect [74]. The defects in the FE layer and the FE-CdS interface provided the low potential site for storing charges which affects the distribution of the charges in the active channel, just like floating gates. On the other hand, the typical ferroelectric memory clockwise loop in Figure 10b indicated that the ferroelectric memory played the dominant role at low temperature. The charge-store memory effect competed with ferroelectric memory as the temperature varied. This was demonstrated by the curve at 140K where no evident hysteretic loop was present (Figure 10c). At low temperature, few trapped charges were active, and the charge-store effect became frozen [75]; therefore, the ferroelectric memory effect became dominant, which was proved by the ferroelectric character at 8.5K (Figure 10d). The positive voltage led to − *P*_r_ in the FE layer and an upward band of core, resulting in a higher potential barrier on the core/shell interface which gave rise to a lower conductance and a positive shift of the electric spectra, as shown in Figure 10d. The binary 1 and 0 can then be defined at 8.5 K, respectively.

**Table 1:** The coupling between size effect and fatigue in different FE systems

**Ferroelectric systems**		**Size effect**		**Fatigue behavior**	
**Ferroelectrics**	**Electrode**	**Size effect**	**Samples**	**Fatigue**	**Samples**
BaTiO_3_, SrTiO_3_, BST, PZT	Metal	Yes	BTO/metal, BST/Pt, PZT/Pt, Ni/SrTiO_3_Pt	Yes	BTO/metal, PZT/Pt
BaTiO_3_,SrTiO_3_,BST,PZT	Electrolyte	No	BTO/LiCl	No	BTO/LiF
BaTiO_3_, SrTiO_3_, BST, PZT	Oxide	No	PZT/RuO_2_,BST/IrO_2_, BST/SRO,PZT/LSCO	No	PZT/RuO_2_,PZT/SRO, PZT/YBCO,PZT/LSCO
SBT	Metal	No	SBT/Pt	No	SBT/Pt
Bi_4_Ti_3_O_12_	Metal	Yes	BIT/Ti–Ag	Yes	BIT/Au
Bi_4_Ti_3_O_12_	Oxide	No	BIT/CaRuO	No	BIT/Sb-doped SnO_2_, BIT/SRO
B_i3.25_La_0.75_Ti_3_o_12_	Metal	N/A	N/A	No	Bi_3.25_La_0.75_Ti_3_O_12_/Pt

#### *CNT-based FeFET*

The performance of the oxide NW-based FeFET is predetermined by the material properties, such as the intrinsic defects and poor field-effect mobility*.* As a flexible and high carrier mobility material with no dangling bond, the carriers in the carbon nanotube (CNT) can realize 1D near-ballistic transport at room temperature [76,77], which is the inherent property that is absent in the traditional oxide NWs [36]. Due to the decrease of the density of states over the increasing energy, the same amount of carriers can induce a more intensified shift of Fermi level than in traditional oxide NWs [37]. CNT therefore has attracted more and more attention with new researches focusing on fabricating CNT-based FET in the past decades [78,79]. With the narrow bandgap of 0.5 eV, the depolarization field is suppressed in CNT, which supplies a much more stable remnant polarization than the traditional oxide NWs. Thus, the enhancement of performance can be obtained from the CNT-based FeFET memory device. However, there still exist many intrinsic flaws in the fabrication of FeFETs. For example, the defects on the interface between the FE layer and single-wall carbon nanotube (SWCNT) can trap charges and hence lead to deterioration of polarization. In addition, the temperature-dependent charge-store memory effect is not controllable as the amount and the distribution of the defects are uncontrollable [80]. Hence, controlling the ‘floating gates’ distributed along the SWCNT channel has been proved difficult.

**Figure 11:**
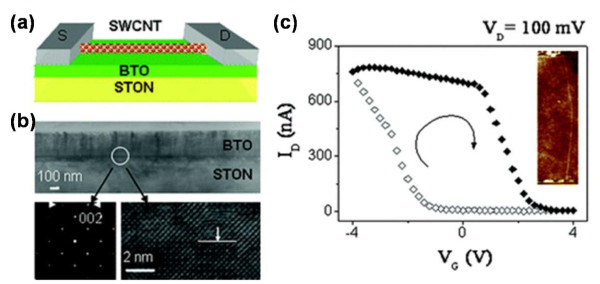
**Schematic sketch and TEM images of the CNT FeFET and the*I***_**D**_**-*V***_**G**_**transfer characteristics.** (**a**) Schematic sketch of the fabricated CNT FeFET. (**b**) Structural characterization of BaTiO_3_ thin films deposited on STON substrates by TEM, indicating the coherent epitaxial growth of the BaTiO_3_ thin film with respect to the STON substrate. (**c**) Typical *I*_D_-*V*_G_ transfer characteristics of the CNT FeFET made of SWCNT with 600 nm in length. The arrows indicate a clockwise hysteresis loop.

**Figure 12:**
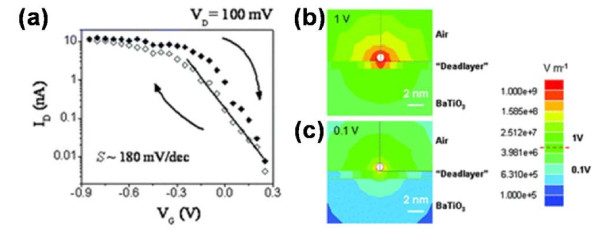
**Transfer characteristics of the device and calculated electric field mappings around the SWCNT channel.** (**a**) Transfer characteristics of the FeFET memory unit with a 300-nm-SWCNT as conducting channel. (**b**,**c**) Calculated electric field mappings around the SWCNT channel at 1 and 0.1 V gate voltages, respectively. The red dashed line in the scale bar indicates the measured coercive electric field of the ferroelectric film.

**Figure 13:**
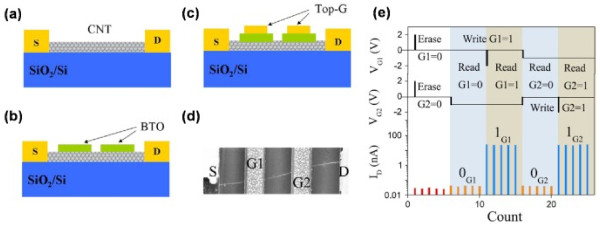
**Scheme of the fabrication of the two top-gated FeFETs assembled on a single nanotube.** (**a**) A CNT FET fortuitously composed of an individual nanotube. (**b**) Coating of amorphous ferroelectric at room temperature onto the top of CNT FET by using PLD. (**c**) After annealing, double top electrodes (G1 and G2) made of Pt were patterned in series onto the deposited ferroelectric films. (**d**) SEM image of the double top-gated CNT FeFET memory. (**e**) The schematic sequential chart for G1/G2 and the programming of the two top-gated CNT FeFET memory.

Based on an excellent FE-CNT interface with few defects in the FE layer, an intrinsic ferroelectric memory FeFET was fabricated by integrating BTO with SWCNT [38]. The moderate preparation process has been carried out to reduce the interface reaction: BTO was pre-prepared on a smooth Nb-doped (001) SrTiO_3_ (STON) substrate by pulsed laser deposition (PLD). Then, the temperate method of spin coating was utilized to deposit SWCNTs onto BTO. Figure 11a showed the schematic diagram of the memory device. The TEM image of the microstructure of the memory device was also displayed in Figure 11b, which revealed a coherent epitaxial growth of BTO on STON. The typical clockwise hysteresis loop was shown in Figure 11c. As we mentioned previously, the *V*_th_ shift was in accordance with the variation of the sweeping region and the initial value. The *V*_th_ values of the device herein were 2.5 V and −1.5 V as *V*_G_ swept upward and downward, respectively, which supplied a wide memory window of 4 V. Thus, when a positive pulse was applied on the active SWCNT channel (assuming a positive voltage of drain-source), the polarization of BTO went from the FE layer towards the SWCNT. When the pulse was released, a high barrier was induced by the downward band bending of SWCNT. Hence, the device was effectively at off state, which could be defined as binary 0. The binary 1 could be defined correspondingly as well. A series of homologous pulses tracking the hysteresis loop could therefore realize a sequence of erases and writes. It should be noted that a compromised hysteresis loop has been observed when the gate voltage was below 1 V, as shown in Figure 12a, whereas the coercive voltage of the FE layer herein was about 2 V. To further investigate this interesting behavior, a theoretical simulation was performed. As shown in Figure 12b,c, the electric field at the interface between the SWCNT and FE layer was far more than *E*_C_ caused by the ultrathin SWCNT; thus, the device could still remain valid at the gate voltage of less than 1 V. Therefore, CNT FeFET offers great potential for manufacturing low-power consumption NVMDs. For a coherent study of this work, a double-gate SWCNT FeFET (a two-bit memory device) was fabricated with the similar technology roadmaps. The schematic maps of the fabrication are shown in Figure 13a,b,c [38]. As a p-type intrinsic SWCNT FeFET memory device, with *V*_DS_ = 10 mV, the device was turned off when a positive gate pulse (2 V) was applied on both gates, which corresponded to the program process of erasing the information stored to binary state 00. The binary information of 01 could be written into an individual FeFET by applying a low bias of −0.5 V on gate 2, which was lower than the threshold voltage yet not enough to induce an efficient polarization in the FE layer. As a result, gate 1 became off (binary 0 state) and gate 2 became on (binary 1 state). The whole program process is shown in Figure 13e, which exhibits a high mobility of approximately 10^3^ cm^2^ V^−1^ s^−1^ and an ultrahigh integration density of over 200 Gbit/in.^2^. This is highly desirable for the practical applications.

#### *Graphene-based FeFET*

Unlike the traditional semiconductor, graphene does not have bandgap. Therefore, the graphene-based FET usually has poor on/off ratio at room temperature [81,82]. Although it has no advantages for digital switches, its high carrier mobility and excellent transconductance make it an ideal material for the radio frequency analog electronics in the logic integrated circuit [83-86]. The high carrier mobility also makes it a promising candidate for the next-generation ultrafast NVMDs [47,87]. Moreover, the enhanced interfacial coupling makes the performance of the graphene-based memory device much more elevated [43,46].

**Figure 14:**
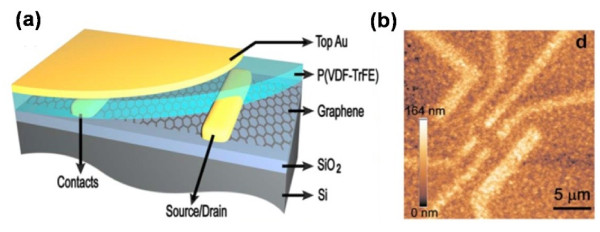
**Schematic diagram and AFM image of a finished memory device.** (**a**) Sample geometry of a finished memory device. (**b**) AFM image of the memory device. The contrast comes from the slightly different crystallization of PVDF-TrFE on SiO_2_, graphene, and Au electrodes, respectively.

**Figure 15:**
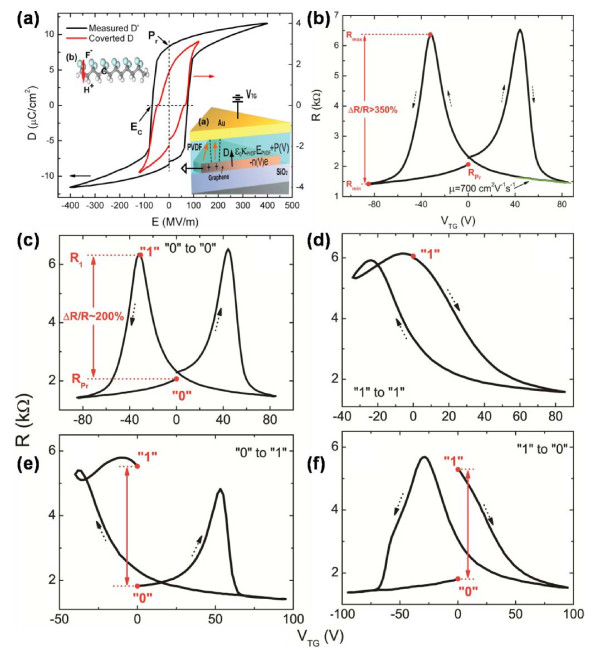
**Electrical switch characteristics of the fabricated device.** (**a**) Resistance hysteretic loop. The black curve represents the experimentally measured D of the PVDF-TrFE thin film with similar thickness. Inset (**a**): the electric displacement continuity equation at ferroelectric-graphene interface. Inset (**b**): a polarized PVDF-TrFE molecule. Cyan, gray, and white atoms represent fluorine, carbon, and hydrogen, respectively. (**b**) Electric hysteresis loop. R is used as a function of V_TG_ for the graphene-ferroelectric sample. From the linear part of this curve at high voltage, the charge carrier mobility is estimated to be 700 cm^2^ V^−1^ s^−1^, taking κ_PVDF_ = 10. (**c**) Switching from 0 to 0 state in graphene-ferroelectric memory by a full loop sweep of V_TG_ (±85 V). (**d**) Switching from 1 to 1 state by an asymmetrical loop sweep of V_TG_ from (85 to −34 V). (**e**) Switching from 0 to 1 state. (**f**) Switching from 1 to 0 state.

The graphene-based FeFET was fabricated using graphene as active channel (Figure 14a) [43]. A 700-nm-thick FE layer of poly(vinylidene fluoride-trifluoroethylene) (PVDF-TrFE) was then spin-coated on the graphene as the top gate. The atomic force microscopy (AFM) image (Figure 14b) showed that the FE layer has formed a continuous thin film. With the switchable polarization of PVDF-TrFE, a resistance hysteretic loop with double peaks was obtained in Figure 15a, due to the induced doping in graphene caused by the flipping electric dipoles. The binary states of 0 and 1 could then be defined as the minimum and the maximum values of *R*, respectively. With the closed hysteretic loop, the program processes could be realized by sweeping *V*_G_ in the specific direction. Regardless of how the binary state transits, the program processes could be realized by following a full loop, as shown in Figure 15c,d,e,f. The graphene-based FeFET featured a high carrier mobility of 200,000 cm^2^ V^−1^ s^−1^ and a reading speed as fast as 10 fs. It should be further noted that the pronounced Δ*R*/*R* exceeded 200 %, which was essential for the retention time and fatigue resistance. These unique characteristics of graphene-based FeFET offer excellent potential in the applications of ultrafast FeFET-based NVMDs.

### Challenges and improvements

**Figure 16:**
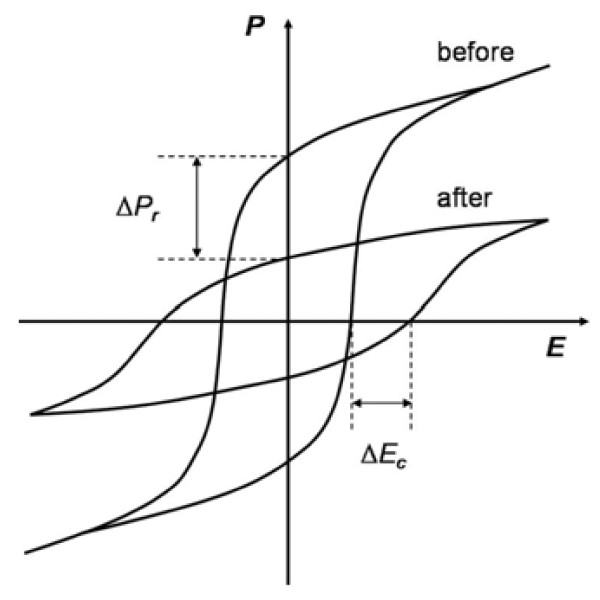
**Hysteresis loops before and after bipolar fatigue.** The loops show decrease in polarization and sometimes an increase in coercive field (particularly for ceramics).

In the previous sections, we have introduced several excellent researches and their potential applications in the domain of NVMDs. However, several inherent flaws have hindered its practical deployment, such as the endurance, fatigue, and retention time [88-91]. Theoretically, the characteristics of ferroelectric would not change. Nevertheless, the visualized experimental transformation of the hysteretic loop of the FE layer revealed that after a number of repetitive bipolar switching cycles, *P*_r_ decreased and *E*_C_ increased [92] (Figure 16). As a result, the smaller *P*_r_ may not be able to induce enough carriers in the active channel and would lead to difficulty in distinguishing the binary signal 0 and 1, which consequently stops the memory device. Moreover, a larger *E*_C_ means that a higher bias is required to switch the device. More researches therefore have been done to overcome these problems, including the introduction of new technologies and new materials to get enhanced performance.

#### *Oxide conductor as electrodes*

According to the model proposed by Dawber [93] and Scott [94], oxygen vacancies in ferroelectric films are believed to be able to impact the fatigue. Due to the local phase decomposition of ferroelectric and the oxygen vacancy migration towards the FE-electrode interface, accumulating and forming a pin structure, the remnant polarization is dramatically decreased [95]. In the typical example of PZT, the oxide conductive materials were utilized as the electrodes [96], which effectively blocked the diffusion effect in each interface. The crystalline structures therefore were not corroded, showing no fatigue behavior. The size effect was associated to the fatigue behavior as well. Table 1 shows a few ferroelectric systems and the characteristics of size effect in relation to fatigue behavior [97].

#### *Insert buffer*

**Figure 17:**
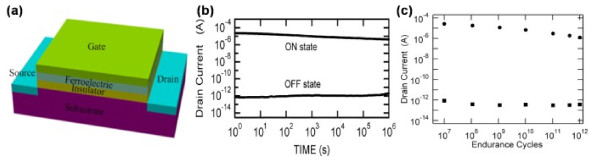
**The schematic structure and memory performance of the device.** (**a**) Pt-SrBi_2_Ta_2_O_9_-Hf-Al-O-Si MFIS FET. (**b**) Data retention characteristic of the Pt-SrBi_2_Ta_2_O_9_-Hf-Al-O-Si FET. After application of the poling voltages ±6 V, the drain currents of both on and off states were measured as a function of time. *V*_keep_ =1.7 V and *V*_D_ =0.1 V. (**c**) Endurance cycle performance of the Pt-SrBi_2_Ta_2_O_9_-Hf-Al-O-Si FET. The applied switching cycle is shown in the inset. The drain currents of the on- and off- states at *V*_G_ = 2 V and *V*_D_ = 0.1 V were measured between multiple cycles.

After a number of switch cycles, the carriers in the semiconductor may inject into the FE layer, which deteriorated the dielectric constant of the FE layer. Moreover, the reduction of remnant polarization was observed after modulating the *V*_G_, as proved by extensive experiments [98-100]. Therefore, a high *κ* buffer was introduced between the FE layer and semiconductor. On one hand, the buffer acted as the diffusion barrier to prevent the ferroelectric from being deteriorated [101]. On the other hand, though the superposed layers were equivalent to two serial capacitors, it made the voltage at the FE layer only slightly smaller than the initial gate voltage [53]. The representative work has been carried out in the early days, which inserted a 13-nm-thick buffer insulation layer of (HfO_2_)_0.75_(Al_2_O_3_)_0.25_ between p-type Si and a 400-nm-thick FE layer of SBT [14]. Figure 17a shows the schematic structure of the device. As shown in Figure 17b,c, it retained an on/off ratio of more than 10^6^ even after 12 days and endured 10^12^ cycles with no changes. Due to the superior retention and endurance, considerable researches have been done to exploit this for practical applications.

#### *Reduction of interfacial states*

**Figure 18:**
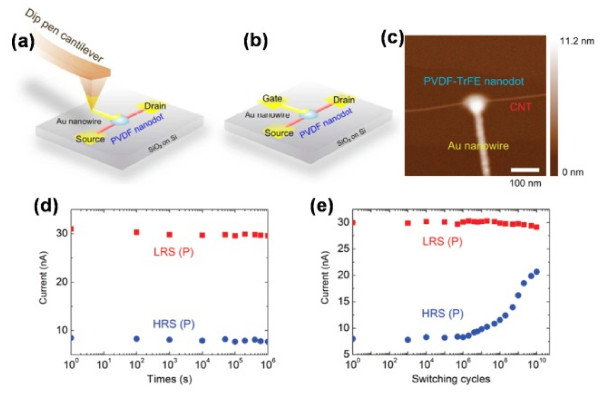
**Schematic illustration of DPN method, the AFM image, and memory performance of the fabricated device.** (**a**) Preparation of an Au-metal gate using AuCl_4_ solution with a writing speed of approximately 300 nm/s by the DPN method. (**b**) A CNT-based nonvolatile memory device made of a CNT channel, a nanostructured PVDF-TrFE-gate insulator, and an Au-metal gate. (**c**) AFM image of the CNT-based nonvolatile memory device, entirely composed of nanostructured elements. (**d**) Two retained *I*_DS_ states plotted as a function of the relaxation time. (**e**) Fatigue-test result of the PVDF-TrFE-based FET memory device.

The scientific experiments have demonstrated that the interface quality of the device is essential for the fatigue behavior [102,103]. The reduced interaction is beneficial for the fatigue resistance, which was demonstrated by the researches executing a post annealing to obtain enhanced performance [19]. Many new technologies and new materials were also introduced to enhance the fatigue resistance, such as the position controllable dip-pen nanolithography (DPN) technology [104]. In this work, PVDF-TrFE was used as the ferroelectric gate. Unlike the inorganic ferroelectric, organic ferroelectric (PVDF-TrFE) has temperate chemical affinity and lower interfacial tension towards the CNT channel, which led to fewer defects in the interface. Figure 18a,b shows the visualized schematic technology maps of the fabricating process, and Figure 18c was the AFM image of the CNT-based nonvolatile memory device. The introduction of nanodot ferroelectric gate (approximately 9.2 nm) realized a high integration density of the memory device, with the bistable state remarkably well retained at up to 10^6^ s. Moreover, as shown in Figure 18d,e, even after approximately 10^10^ switching cycles, the two states were still distinguishable, demonstrating great performance.

## Conclusions

**Figure 19:**
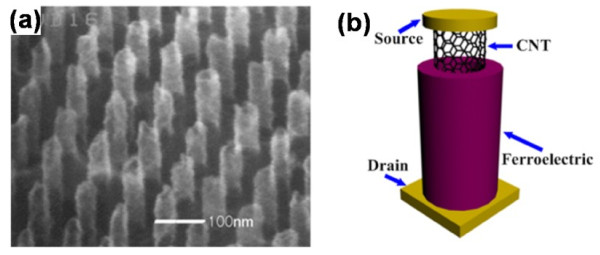
SEM image of vertically aligned CNTs (a) and suggested device architecture of surround-gated vertical CNT-based FeFET (b).

In this paper, we explain the operating principles of FeFET and review several excellent researches on the integration of semiconductor materials with ferroelectric to achieve agreeable memory performance. The advantages of the nonvolatility and NDRO process make FeFET ideal for memory applications. With the development of material fabrication technologies, the non-planar ferroelectric nanostructures such as FE NWs [105-107], nanotube [108-110], and NPs [108] have been prepared successfully. Although the capacity of the first FeRAM had only a 256-bit density [111], with the incorporation of the modern semiconductor technology, ferroelectric nanostructures with much higher integration density have been integrated in a large scale [112,113]. The integration density can be further enhanced with new technologies and/or new device structures. Based on the current achievements on the controllable and selective growth of CNT arrays, as shown in Figure 19a, we suggest a new FeFET architecture by integrating CNT arrays with ferroelectric to further enhance the integration density of the memory devices. While current FeFET advancements have supplied potential routes to overcome the scale limitations and economic challenges, the fatigue and retention time remain as the main challenges hindering the practical applications. It still will be a long way to go to realize the mass commercial production.

## Competing interests

The authors declare that they have no competing interests.

## Authors’ contributions

XL wrote and revised the manuscript. YL and WC suggested many helpful and interesting issues for improving the review paper. JL revised the paper thoroughly. LL drafted and revised the manuscript. All authors read and approved the final manuscript.
